# Progress in the Practice of Medicine

**Published:** 1874-10

**Authors:** Norman Bridge

**Affiliations:** Lecturer on Principles and Practice of Medicine in Rush Medical College


ARTICLE III.—*Progress in the Practice of Medicine.* By NORMAN BRIDGE, M.D., Lecturer on Principles and Practice of Medicine in Rush Medical College.

1. The Blister Treatment of Rheumatism. By Dr. T. R. PEACOCK, St. Thomas Hospital, London.

2. The Use of Large Injections of Water for Intestinal Obstruction. By Dr. ROBERT BATTEY, of Atlanta, Ga. (*Atlanta Med. & Surg. Jour.*)

3. Local Treatment of Lung Cavities. By Dr. J. H. HUTCHINSON. (*Philad. Med. Times.*)

4. Œsophagismus. By Dr. A. W. FOOT, Ireland. (*Irish Hosp. Gazette—Am. Jour. Med. Sciences.*)

5. Angioleucitis of the Lung. By Dr. REYNAUD. (*L'Union Med.—Am. Jour. Med. Sciences.*)

6. Guarana in Chronic Rheumatism. By Mr. A. E. RAWSON. (*Irish Hosp. Gaz.—Am. Jour. Med. Sciences.*)

7. Therapeutics of Gelseminum. By Dr. W. C. HULL. (*Med. & Surgical Reporter.*)

1. Dr. P. has used the blister treatment for acute rheumatism since 1865. He has a high opinion of it when vigorously applied. The blisters are generally made two or three inches wide, and long enough to encircle the limb. When they have risen, they are dressed with linseed meal poultices for several days. Usually, the patients do not complain of the blisters, nor is there any serious inconvenience from them; but as soon as the drawing is accomplished there is a marked amelioration in the swelling, pain and constitutional disturbance. The cases most eligible for treatment by this means, are those where several joints are involved and the suffering and constitutional disturbance great. Three to six blisters are applied at once near the affected joints; they are repeated as exacerbations occur, and are applied to joints as they are successively involved.

He believes this treatment often curtails the length of the disease and prevents internal complications. He regards the blisters as less depressing than the alkalis and other constitutional medication, except tonics which he often gives.

2. After quoting from authors advocating large injections of water for intestinal obstruction, and from his own previous writings to the same purpose, Dr. B. advances the theory, which he claims as original, that fluid can be not only forced through the ileo-cæcal valve into the small intestine, but that it may be forced through the whole length of the alimentary canal and out the mouth. Two cases are reported where this unmistakably took place, the quantity of tepid water used being in each case little less than twenty-four pints. In one case, turpentine soap was mixed with the water, and the patient protested she could taste the soap in the clear fluid vomited as the injection was being passed. He had, with Prof. Johnson experimented on the cadaver by injecting cold water into the rectum. "It found no obstacle to its onward progress at the ileo-cæcal valve; it passed easily through the small intestines, stomach and œsophagus and out the mouth."

He gives the large injections advised with a syringe that throws a continuous stream—it being operated slowly. He would have air carefully excluded from the water, believing that air tends to induce sudden discharge of the fluid; and when pain is produced by the injection, he gives anodynes—opium or chloroform, and if necessary he anæsthetizes the patient. He holds this to be altogether the most proper of all means of relief for the various obstructions of the intestines, and hopes to see it supersede the use of the knife in most cases of strangulated hernia.

The novel and distinctive part of his paper is that wherein he shows that *very large* quantities of water may be injected, and to a point far beyond the ileo-cæcal valve, and all *without harm*. This he especially insists on, and combats the doctrine of Dr. Flint, Sr., and others, that large injections of water when pain is produced are hazardous, and are to be used with great caution

3. A good deal has been said and written of late, on the subject of the local treatment of lung cavities by injections into them made by means of a trochar and canula plunged between the ribs and through the lung tissue.

Prof. Mosler, of Germany, and Dr. Wm. Pepper, of Philadelphia, have recently treated a number of cases in this way, and speak encouragingly of the treatment. Prof. M. says his experiments prove that the lung is "more tolerant of external manipulation, and that these external attacks are more easily executed and less dangerous than has been heretofore believed."

Dr. P. thinks, considering the almost hopeless nature of some of these lesions, the treatment should be tried patiently.

Dr. Hutchinson, however, by quotations from the records, shows that the operation has been at three or four different brief periods quite popular, and that it has been in each instance so completely forgotten that it has been as many times advanced as new. This, he thinks, makes a strong presumption against the measure. He holds it to be unadvisable for the following additional reasons: Punctured wounds of the lung in a consumptive will not do well because a similar injury to a healthy lung does well. Any inflammation excited by a wound of the lung is likely to eventuate in caseous degeneration. Injections are not proper treatment for phthisical cavities; they tend to excite inflammation, whereas there should be an attempt to allay it. There is more or less danger of a discharge being by this treatment emptied into an unadherent pleural cavity. He believes with Dr. Hughes Bennett, that all operative interference in phthisis has been a "uniform failure."

4. Dr. Foot reports four cases of this disease; three in men and one in a woman. The cases were alike in occurring suddenly; in there being more or less intermittent dysphagia, and this more easily excited by solid food, by cold and by sour substances; in the occurrence of hiccough and œsophageal vomiting. The patients were all nervous people and the symptoms were clearly beyond their control, they were not feigned. In none was there evidence of organic disease or hysteria. Cases of œsophagismus appear to be most common in males, while regurgitation proper is most frequent in females.

Dr. MacSwiney (present at the meeting) had seen three similar cases, all in men, and all recovered. In one case there was enormous dilatation of the œsophagus above the seat of spasm. Antispasmodics were alone valuable, and hydrocyanic acid was most efficacious of all.

Dr. Kennedy had seen five cases; all recovered.

5. Dr. R. has coined this new word, *angioleucitis.* He applies it to a lesion heretofore not described, which consists in a vascular turgescence of the lymphatic vessels of the lungs. While this disorder has some relation with cancer, especially of the stomach, he believes a true case of angioleucitis may occur unconnected with carcinoma. The affection is grave, especially when it occurs as a complication of other organic disease.

He reports two cases illustrative of his description of the disease.

6. Mr. R. tried guarana on his own person, as an experiment, for severe lumbago. Fifteen grains gave perfect relief, but on a recurrence of the pain a larger dose was required. He has since experimented extensively with the drug on all sorts of patients, and arrives at this conclusion: "Whenever the fibrous sheaths of the nerves or muscles, the fasciæ or tendons, are affected, guarana gives immediate relief, which lasts twelve to twenty-four hours." He believes that a perseverance in the use of the drug, increasing the dose to forty grains, will entirely remove any of the above-mentioned kinds of rheumatism.

7. Dr. H., with an associate, says he has administered gelsemium in a thousand cases. He concludes it is not adapted to inflammatory diseases. In those in which there is active congestion it does injury. "Its therapeutic scope is confined to certain simple forms of fever." "In bilious, catarrhal and gastric fevers of childhood it is nearly a specific." In typho-malarial fever, where the malarial character predominates, it often abates the disease. It should, when administered, be rapidly introduced into the system until the characteristic effects are produced on the organs of vision.

